# Unbiased Proteomics Analysis Demonstrates Significant Variability in Mucosal Immune Factor Expression Depending on the Site and Method of Collection

**DOI:** 10.1371/journal.pone.0079505

**Published:** 2013-11-14

**Authors:** Kenzie M. Birse, Adam Burgener, Garrett R. Westmacott, Stuart McCorrister, Richard M. Novak, T. Blake Ball

**Affiliations:** 1 Department of Medical Microbiology, University of Manitoba, Winnipeg, Manitoba, Canada; 2 Proteomics Group, National Lab for HIV Immunology, Public Health Agency of Canada, Winnipeg, Manitoba, Canada; 3 Mass Spectrometry Core Facility, National Microbiology Laboratory, Public Health Agency of Canada, Winnipeg, Manitoba, Canada; 4 Section of Infectious Diseases, University of Illinois, Chicago, Illinois, United States of America; 5 Department of Medical Microbiology, University of Nairobi, Nairobi, Kenya; 6 National Lab for HIV Immunology, Public Health Agency of Canada, Winnipeg, Manitoba, Canada; University of Cape Town, South Africa

## Abstract

Female genital tract secretions are commonly sampled by lavage of the ectocervix and vaginal vault or via a sponge inserted into the endocervix for evaluating inflammation status and immune factors critical for HIV microbicide and vaccine studies. This study uses a proteomics approach to comprehensively compare the efficacy of these methods, which sample from different compartments of the female genital tract, for the collection of immune factors. Matching sponge and lavage samples were collected from 10 healthy women and were analyzed by tandem mass spectrometry. Data was analyzed by a combination of differential protein expression analysis, hierarchical clustering and pathway analysis. Of the 385 proteins identified, endocervical sponge samples collected nearly twice as many unique proteins as cervicovaginal lavage (111 vs. 61) with 55% of proteins common to both (213). Each method/site identified 73 unique proteins that have roles in host immunity according to their gene ontology. Sponge samples enriched for specific inflammation pathways including acute phase response proteins (p = 3.37×10^−24^) and LXR/RXR immune activation pathways (p = 8.82×10^−22^) while the role IL-17A in psoriasis pathway (p = 5.98×10^−4^) and the complement system pathway (p = 3.91×10^−3^) were enriched in lavage samples. Many host defense factors were differentially enriched (p<0.05) between sites including known/potential antimicrobial factors (n = 21), S100 proteins (n = 9), and immune regulatory factors such as serpins (n = 7). Immunoglobulins (n = 6) were collected at comparable levels in abundance in each site although 25% of those identified were unique to sponge samples. This study demonstrates significant differences in types and quantities of immune factors and inflammation pathways collected by each sampling technique. Therefore, clinical studies that measure mucosal immune activation or factors assessing HIV transmission should utilize both collection methods to obtain the greatest representation of immune factors secreted into the female genital tract.

## Introduction

Mucosal secretions provide a barrier against invading pathogens and microorganisms. In the case of HIV-1, heterosexual intercourse is the main route of new infections [1], making the mucosa of the female genital tract (FGT) the first site of contact for male to female HIV-1 transmission. This mucosal surface is complex, and contains an abundance of soluble innate immune factors that are important for HIV-1 acquisition. Such factors include RANTES [2] MIPα, MIPβ, SLPI [3], Elafin [4], [5], LL-37 [6], α/β-defensins [7], [8], Lysozyme, Lactoferrin, Calprotectin, Histone H2A [9], Cystatins, Serpins [10] as well as many other anti-proteases [11]. The composition and balance of these factors may influence susceptibility to HIV-1, as shown in studies of HIV-exposed seronegative (HESN) individuals and individuals who succumb to infection [4], [11]–[16]. These factors may have an impact on local viral replication, establishing the viral load set point and the rate of disease progression [17]. Also, follow-up studies to determine the correlates of protection in HIV vaccines that have shown promise such as the Thai RV144 trial [18] have emphasized the importance of mucosal immune responses in reduced acquisition [19]. Therefore, it is critical that these factors are properly measured to understand early events in HIV pathogenesis and transmission.

Recent clinical trials have indicated that increased immune activation in the FGT has been attributed to increased risk of HIV-infection. The importance of mucosal inflammation was exemplified by the failure of the detergent microbicide, nonoxynol-9, which increased HIV-infection risk and was associated with an increase in inflammation status in the FGT [20]. Furthermore, the lack of efficacy in the Centre for AIDS Programme of Research in South Africa (CAPRISA-004) microbicide trial may also have been attributed to increased baseline immune activation and pro-inflammatory cytokine production [16], [21]. However, as the biological determinants of FGT inflammation and the immune pathways important for HIV-susceptibility have not yet been defined, defining techniques and protocols to efficiently and accurately monitor a broad range of factors involved with inflammation and immune activation in the mucosal compartment will be essential for future clinical trials and the development of future intervention technologies.

The most commonly used techniques to sample the FGT mucosa involve the use of cervicovaginal lavages and/or Weck-Cel cervical sponges, which are largely standardised, inexpensive and minimally invasive [22]–[24]. Each method collects secretions from different compartments of the FGT. Cervicovaginal lavages are designed to collect secretions primarily from the lower FGT, which includes the ectocervix and the vaginal vault, and Weck-Cel cervical sponges are designed to collect secretions primarily from the upper FGT, which includes the endocervix and the endometrium. However, it is unknown which technique and/or site sampled is most informative, and most relevant with respect to inflammation and the collection of immune factors. Although previous studies examining sampling techniques have noted some differences between the methods [23], these have been limited to a small list of pre-defined immune factors such as Secretory leukocyte peptidase inhibitor (SLPI), Human neutrophil peptides (HNP) 1–3, and Lactoferrin, despite the need to measure many other mucosal factors important in HIV-1 susceptibility and inflammation. Therefore, a comprehensive examination is warranted to better evaluate these collection techniques and the sites primarily sampled by each.

This study uses an unbiased systems biology proteomics approach via tandem mass spectrometry to compare FGT secretions using different collection methods to determine the qualitative and quantitative differences in the captured immune factors. Proteomics confers advantages to other methods as it can provide a global view of biological factors in a given sample and has been effective in monitoring the often difficult to measure, mucosal immune responses [11], [12], [14], [25]. The mucosal secretions collected by paired cervicovaginal lavages (CVL) and Weck-Cel cervical sponges (CER) were compared from 10 healthy women. In this study, we show considerable differences in both the number and abundance of mucosal factors identified in either collection method due to both the site of collection and sampling technique.

## Materials and Methods

### Ethics Statement & Study Population

Study participants were women at low risk for HIV acquisition. To participate they had to meet the following criteria: never used crack, never exchanged sex for money, drugs or shelter, no more than one sexual partner in the last 6 months, no more than 5 sexual partners in the last 5 years, and no history of sexually transmitted infections. Study participation required written, informed consent, and was approved by the human subjects committee of the University of Illinois at Chicago. All of the participants underwent testing for Bacterial vaginosis, *Trichomonas vaginitis*, *Neisseria gonorrhoeae,* and *Chlamydia trachomatis* at the time of collection. Samples positive for any of these tests were excluded from the study. Participants included in this study were between the ages of 18 to 27 and were not on any form of hormonal contraception. Of the ten individuals included in this study, 8 were in their luteal phase and 2 were in their follicular phase of the menstrual cycle, and 2 out of the 10 had vaginal sex within 24 hours of sample collection. These factors should not confound the data obtained from this study as both CVL and CER samples were collected from the same individual at the same time. Any effect of these factors will be represented equally in sampling techniques.

### Cervicovaginal Lavage (CVL) and Weck-Cel Cervical Sponge (CER) Sample Collection

For all participants, a speculum was inserted into the vagina and the cervix was located. Four cotton tipped brushes were used to swab the posterior, lateral, frontal and ectocervical areas of the FGT for standard sexually transmitted infection testing. Next, a Weck-Cel cervical sponge was inserted into the cervical os and allowed to sit for one minute to allow for the collection of secreted factors from the upper FGT, the endocervix and the endometrium. The Weck cells were weighed in spin-x tubes before collection and after collection to determine the volume collected. The volume eluted from each sponge was ∼100–200 µL. After the sponge’s removal, the cervicovaginal lavage sample was obtained by washing 10 cc of normal saline over the vaginal vault and ectocervix. The saline lavage solution was then redrawn using the same syringe of which it was instilled. All samples were immediately stored on ice and subsequently frozen at −80°C within 1 hour of sample collection.

### Protein Digestion and Preparation for MS Analysis

CVL and CER sample protein content was measured by standard BCA protein assay (Novagen). One hundred micrograms from ten CVL samples and ten matching CER samples obtained from the same individual were each individually denatured with urea exchange buffer (8 M Urea GE HealthCare, 50 mM HEPES Sigma, pH 8.0) for 20 minutes at room temperature placed into Nanosep filter cartridges (10 kDa). After centrifugation samples were treated with 25 mM dithiothreitol (Sigma) for 20 minutes, then 50 mM iodoacetamide (Sigma) for 20 minutes, and washed with 50 mM HEPES buffer. Trypsin (Promega) was added (2 µg/100 µg protein) and incubated at 37°C overnight in the cartridge. Peptides were eluted off the filter with 50 mM HEPES, and were dried via vacuum centrifugation. The samples were then cleaned of salts and detergents by reversed-phase liquid chromatography (high pH RP, Agilent 1200 series micro-flow pump, Water XBridge column) using a step-function gradient such that all peptides elute into a single fraction for each sample. The fractions were then dried via vacuum centrifugation and kept at −80°C until analyzed by mass spectrometry.

### Mass Spectrometry Analysis

The same amount of total protein from each sample was then prepared for label-free tandem mass spectrometry analysis. Fractions were re-suspended in 2% acetonitrile (Fisher Scientific), 0.1% formic acid (EMD Canada) and injected into a nano-flow LC system (Easy nLC, Thermo Fisher) connected inline to a LTQ Orbitrap XL (Thermo Fisher) mass spectrometer. All spectra were processed using Mascot Distiller v2.3.2 (Matrix Science), and database searching was done with Mascot v2.3 (Matrix Science). Searches were performed against UniProtKB/SwissProt (2012-05) Human (v3.87) database. Label-free protein expression levels based on MS peak intensities were calculated using Progenesis LC-MS software (v4.0 Nonlinear Dynamics). The relative abundance ratios of these proteins were calculated by dividing each value by the average intensity across all samples. Statistical analysis of protein expression was performed by one-way analysis of variance (ANOVA). Complete details of liquid chromatography, mass spectrometry instrument settings, data generation and the complete protein expression data set can be found at our public database (www.corefacility.ca/proteomics/data/burgener/pubs/PLOS).

### Clustering and Pathway Analysis

Cluster analysis was performed using Cluster software, version 3.0, and data was visualized using TreeView software, version 1.1.5 (13). CVL samples and CER samples were grouped separately in Perseus, version 1.3.0.4 (Max Planck Institute of Biochemistry), and all proteins were filtered based on p-values as determined by One-way ANOVA (p<0.05). Clustering of differentially abundant proteins was generated by unsupervised centroid linkage hierarchical clustering using Pearson correlation coefficient as the distance metric. Each main dendrogram branch specific to the enrichment of proteins based on collection method from the cluster analysis was analyzed by Ingenuity Pathway Analysis (Ingenuity Systems, www.ingenuity.com, Mountain view, CA). The association between proteins in the dataset and the canonical pathways in the Ingenuity Pathway Knowledge base was measured as a ratio of the number of molecules from the data that maps to a pathway divided by the total number of molecules known to map the canonical pathway. A right-tailed Fisher’s Exact Test (with Benjamini-Hochberg multiple testing correction) was used to calculate the p-value of the probability that the association between each protein in the dataset and canonical pathway is random. Pathways with p-values <0.05 and at least 2 proteins selected were considered as potential pathways associated with each branch in the cluster analysis. Functional annotation of the proteins differentially expressed by either collection method was performed using DAVID Bioinformatics Resources (6.7); biological functions were determined based on gene ontologies. Graphical representation of innate factors was constructed using GraphPad Prism (5.0a, GraphPad Software) using Mann-Whitney statistical tests.

## Results

### Mass Spectrometry Analysis of Mucosal Proteins Identifies Large Numbers of Proteins Specific to Cervicovaginal Lavages and Endocervical Sponge Samples

An unbiased, label-free proteomics approach was used to identify and quantify proteins recovered using the two most commonly used collection methods of mucosal secretions from the FGT. To allow a direct comparison, matching CER and CVL samples were collected from 10 healthy women donors. Protein concentration varied between collection methods as CER samples had an average of 2.65 µg/µL, and CVL samples had an average of 0.63 µg/µL. Based on the determined concentrations, equal amounts of total protein from each sample was then digested, processed and individually analyzed by mass spectrometry as outlined in the Materials and Methods.

**Figure 1:**
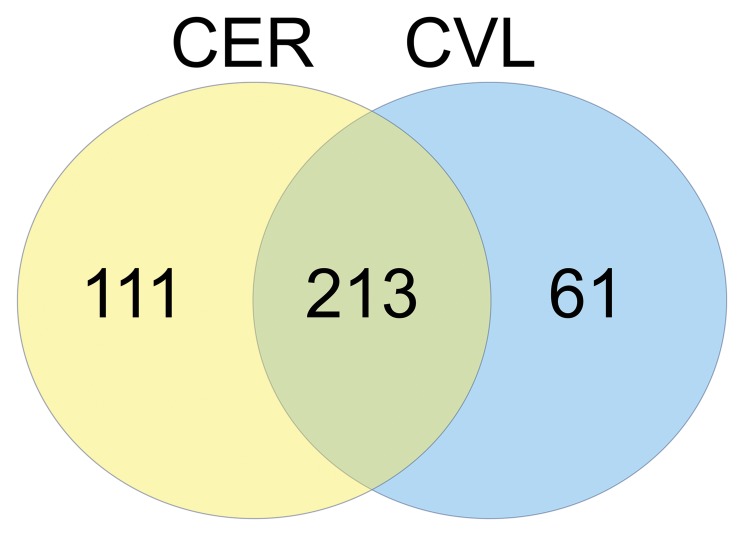
Venn diagram of the number of unique proteins collected via either sampling technique. Weck-Cel cervical sponge (CER) method (111), the cervicovaginal lavage (CVL) method (61), and the number of overlapping proteins identified by both methods (213).

A total of 385 unique proteins were identified with high confidence. The relative abundance ratios of these proteins were calculated by dividing each value by the average intensity across all samples. A complete list of proteins and their relative abundance based on sample type are available as supplemental information (Table S1). A Venn diagram was constructed to illustrate the overlap and the number of unique proteins identified by each method (Figure 1). While 213 unique proteins were identified by both sampling techniques, 111 were identified solely in the CER samples, and another 61 unique proteins were exclusive to CVL samples. Of the 213 unique proteins common to both sampling techniques, 73 were found to have roles in immune functions according to their gene ontology.

**Figure 2:**
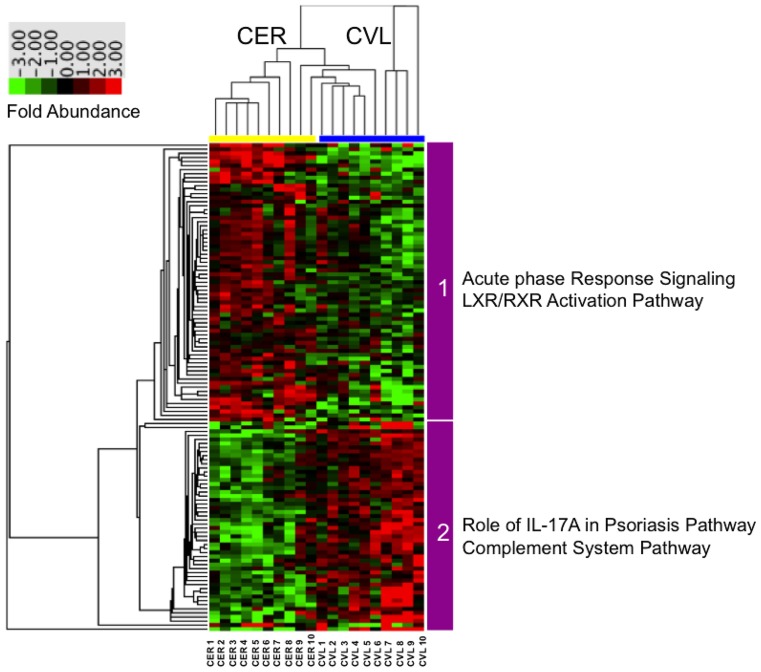
Protein abundance patterns differentiate proteins identified and recovered by both collection methods. the Weck-Cel cervical sponge method (CER) and the cervicovaginal lavage method (CVL) (Proteins shown (121) were identified as differentially abundant by one-way ANOVA (p<0.05)). Clustering of proteins was generated by unsupervised centroid linkage hierarchical clustering using the Pearson correlation coefficient as the distance metric. Protein abundance levels are shown in colour, with red indicating overabundant proteins and green indicating underabundant proteins compared to the median of all samples for either collection method.

To visually map the differences in protein abundances found in common across each technique, hierarchical clustering analysis was performed on the dataset. The cluster software utilizes algorithms to calculate relatedness between study participants and between protein expression patterns, and then constructs a representative dendrogram. For example, proteins of the same family, or pathway, will group more closely together as compared to proteins from another family. Proteins that showed a differential abundance (p<0.05 as determined by one-way ANOVA) (121) were analyzed using a correlation – uncentered similarity metric and unsupervised centroid linkage hierarchical clustering to produce a hierarchical clustering heat map (Figure 2). A clear pattern emerged in the dendrogram based on the sample collection method used. For example, branch 1 (top branch) clustered 68 proteins that were overabundant in the secretions collected by the CER samples, and branch 2 (bottom branch) clustered 50 proteins that were overabundant in the secretions collected by CVL. The cluster analysis was able to clearly discriminate between sampling techniques by protein abundance, which reflects a bias in each technique’s ability to capture specific protein factors, and the types of factors primarily secreted by each compartment of the FGT.

### Site of Collection and Sampling Technique Differentially Enriches for Specific Inflammation Pathways and Functions

The two branches were further analyzed using Ingenuity Pathway Analysis Software to determine the top pathways associated with the proteins identified using each collection method (Table 1). The top two pathways associated with branch 1 (CER-enriched proteins) were immune activation pathways including the Acute Phase Response Signaling pathway with 20 out of 177 proteins of the pathway identified (p = 3.37×10−24), and the Liver X Receptor/Retinoid X Receptor (LXR/RXR) Activation pathway with 17 out of 136 protein identified (p = 8.82×10−22) in this pathway. Less significant associations were found with branch 2 (CVL-enriched proteins) which corresponded to pathways involved in innate immunity including the IL-17A in Psoriasis pathway with 2 out of 13 proteins identified (p = 5.98×10−4), and the Complement System pathway with 2 out of 35 proteins identified (p = 3.91×10−3). This indicates that pathways involved with acute phase responses; immune activation and innate immunity are differentially enriched depending on the site and collection method.

**Table 1:** Protein pathways selectively enriched based on FGT compartment/collection method.

Weck-Cel Cervical Sponge Enriched Pathways (Branch 1)		Cervicovaginal lavage Enriched Pathways (Branch 2)	
Pathway	p-value	Pathway	p-value
Acute Phase Response Signaling (20/177)*	P = 3.37×10^−24^	Role of IL-17A in Psoriasis	P = 5.98×10^−4^
LXR/RXR Activation (17/136)	P = 8.82×10^−22^	Complement System (2/35)	P = 3.91×10^−3^

* Denotes the number of protein factors identified in the proteomic dataset out of the total number of listed proteins involved in the pathway based on the Ingenuity software platform.

**Figure 3:**
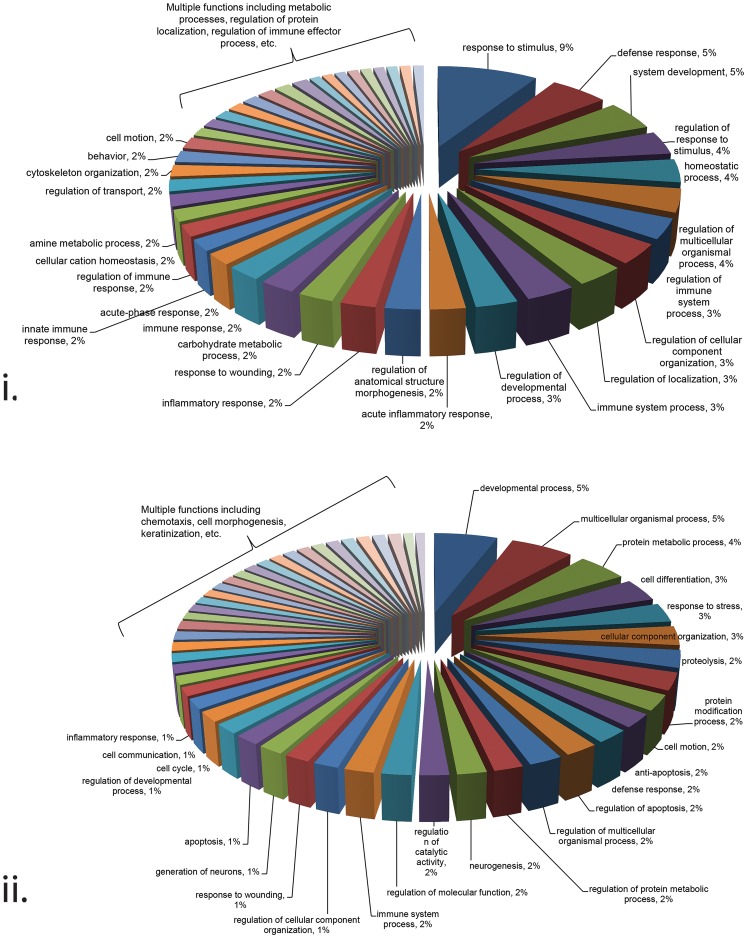
The biological functions of the proteins determined via hierarchical cluster analysis. Proteins associated with Branch 1 (i) and Branch 2 (ii) of the hierarchical cluster analysis according to their gene ontology determined via the functional annotation tool from DAVID Bioinformatics Resources.

The two major branches of hierarchical clustering map were then further grouped using DAVID Functional Annotation Bioinformatics MicroArray Analysis tool [26], [27], which grouped proteins based on their major biological functions according to their gene ontology (Figure 3). Of those proteins found to be overabundant in secretions from the upper FGT collected via CER samples, 28% had immune response functions. Similarly, but to a lesser degree, 8% of the proteins found to be overabundant in secretions primarily from the lower FGT collected via CVL samples had immune response functions. These slight functional differences can partially be attributed to the differences in unique proteins identified by each method/site. For example, specific defense response proteins found in CER samples included Beta-2-microglobulin, Haptoglobin, and Complement component C3 to name a few, whereas the specific defense response proteins identified in CVL samples differed and included proteins such as Calprotectin (S100-A8/A9), Cathepsin G, and Neutrophil defensin-1. This demonstrates that each method and site of collection collects proteins involved in overlapping immune pathways, but each is differentially enriched for specific members of these pathways. Therefore, specific protein recovery is variable based on either collection method and/or the specific compartment of the FGT from which the secretions were primarily collected.

### Antimicrobial and Immune Mediator Expression Varies Considerably in Abundance between CVL and CER Samples

Antimicrobial factors and other immune regulators with described involvement in mucosal immune activation were examined in greater detail due to their involvement with immune activation as well as antiviral defense. These included antimicrobial and HIV inhibitory factors such as SLPI [3], [28], Defensins [7], [8], Lysozyme C [29], Mucins [30], mucosal IgA [31], [32]; antibacterial factors of the S100 protein family [33], protease inhibitors such as Serpins [10], [34], [35], A2ML1 and Cystatins [11], [29]; and proteases such as Cathepsins [36].

**Figure 4:**
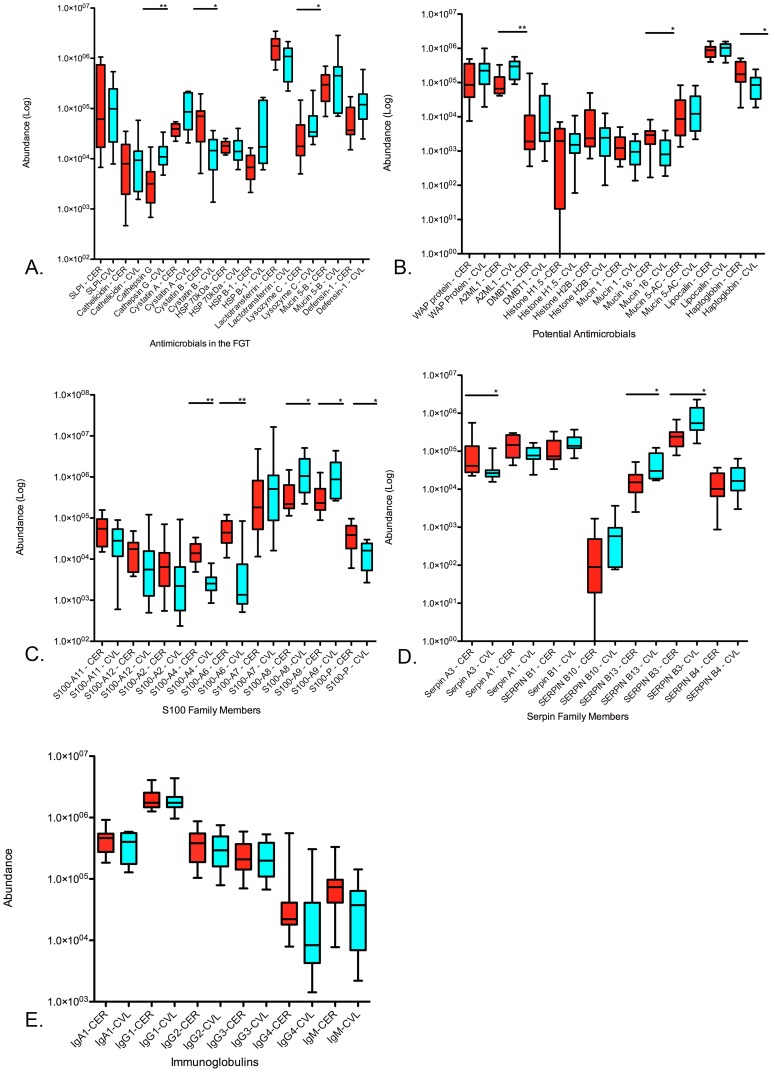
Box plots demonstrating the abundances of various immune factors important for host defense. CER (endocervical sponge, red) and CVL (cervicovaginal lavage, blue). A: General antimicrobial factors, B: Potential antimicrobials, C: S100 calcium-dependent S100 family members, D: Serpin family members, E: Immunoglobulins (heavy chain variants). Significance values are shown as follows: * = <0.05, ** = <0.005 (Mann-Whitney statistical tests).

Antimicrobial factors identified from both sites were quantified and the abundance values compared as shown in Figure 4A. Many of these were found to be significantly different between compartments such as Cathepsin G and Lysozyme C which were found to be overabundant in CVL as compared to CER (3-fold increase, p = 0.002 and 1.7-fold increase, p = 0.04, respectively) and Cystatin B which was found to be overabundant in CER as compared to CVL (4.4-fold increase, p = 0.01). Other potential antimicrobial factors (Figure 4B), such as A2ML1, Haptoglobin, and Mucin 16 were found to be differentially abundant based on sampling technique, with A2ML1 overabundant in CVL (2.7-fold increase, p = 0.004,), and Haptoglobin and Mucin-16 overabundant in CER (2.5-fold increase, p = 0.04 and 2.5-fold increase, p = 0.03, respectively).

Many calcium-binding proteins from the S100 Family were identified in both sites using the different collection methods used in this study (Figure 4C). S100 proteins are ion-binding proteins that are known to be associated with native anti-inflammatory processes [37]–[39]. Certain members were found to be differentially abundant based on sampling technique/compartment, such members include: S100-A4, -A6, -A8, -A9 and -P. Members S100-A4, S100-A6 and S100-P were all found to be significantly overabundant in the CER samples (5.3-fold increase, p<0.0001, 4.8-fold increase p = 0.0007 and 2.8-fold increase, p = 0.01, respectively), and members S100-A8 and S100-A9, also known as Calprotectin were found to be significantly overabundant in CVL (3.8-fold increase, p = 0.01 and 3.6-fold increase, p = 0.01, respectively). Serpins were also recovered by both collection methods, and have recently been described as important factors in HIV-1 susceptibility [12], [15] as they have been shown to be anti-inflammatory proteins and possess inhibitory properties against HIV-1 [10], [34]. Serpin B13 and B3 were significantly overabundant in CVL (2.9-fold increase, p = 0.01 and 3.2-fold increase, p = 0.007, respectively), and Serpin A3 was significantly overabundant in CER (3.4-fold increase, p = 0.03) as shown in Figure 4D. The detection of immunoglobulins was comparable by each method (Figure 4E), but 25% of immunoglobulins detected were only found in CER samples including IgA-2. The statistical distribution of these antimicrobial factors as well as the percentage of participants where an antimicrobial factor was reliably detected (found within +/−1 standard deviation) are shown in Tables 2–6. A complete list of all proteins identified and their basic statistics are available as supplemental information (Table S2), which can be found at our public database. (www.corefacility.ca/proteomics/data/burgener/pubs/PLOS).

**Table 2:** Basic abundance variation of general antimicrobial factors collected via Weck-Cel cervical sponge and cervicovaginal lavage as determined by mass spectrometry.

	CER Protein Statistics				CVL Protein Statistics			
Protein	Mean(x10^3^)	Std. Dev.(x10^3^)	Range(x10^3^)	% detected+/−1SD	Mean(x10^3^)	Std. Dev.(x10^3^)	Range(x10^3^)	% detected+/−1SD
SLPI	346.509	429.916	1056.26	80	155.141	179.572	534.158	80
Cathelicidin antimicrobial peptide	11.484	11.205	34.6626	90	12.694	16.82	56.804	90
Cathepsin G	4.398	4.813	16.4772	90	13.493	8.521	29.214	80
Cystatin-A	39.053	11.028	32.168	70	106.417	78.123	198.986	50
Cystatin-B	68.922	55.165	190.291	70	15.711	11.181	35.01	70
Heat shock 70 kDa protein 1A/1B	17.933	4.937	13.446	70	17.174	10.725	34.228	70
Heat shock protein beta-1	8.104	4.749	14.284	60	57.748	68.91	160.24	70
Lactotransferrin	1830	960.559	2873.91	60	1060	661.284	1923.95	50
Lysozyme C	35.082	42.614	141.767	90	61.211	63.987	210.252	90
Mucin-5B	311.705	215.575	626.387	60	623.761	818.538	2776.57	90
Neutrophil defensin 1	66.262	55.534	156.966	80	158.861	165.313	573.889	90

**Table 3:** Basic abundance variation of potential antimicrobial factors collected via Weck-Cel cervical sponge and cervicovaginal lavage as determined by mass spectrometry.

	Endocervical Sponge Protein Statistics				Cervicovaginal Lavage Protein Statistics			
Protein	Mean(x10^3^)	Std. Dev.(x10^3^)	Range(x10^3^)	% detected+/−1SD	Mean(x10^3^)	Std. Dev.(x10^3^)	Range(x10^3^)	% detected+/−1SD
WAP four-disulfide core domain protein 2	167.994	179.872	481.534	80	275.326	284.568	979.057	90
Alpha-2-macroglobulin-like protein 1	109.763	97.476	289.359	80	294.084	163.065	477.099	70
Deleted in malignant brain tumors 1 protein	22.892	57.77	185.9433	90	23.491	33.482	91.7083	80
Histone H1.5	2.485	2.5	7.019	70	2.745	3.505	10.793	80
Histone H2B type 1-L	10.643	15.853	49.195	90	3.386	3.772	12.5247	90
Mucin 1	1.7	1.473	4.684	80	1.202	0.9715	3.016	80
Mucin-16	3.232	2.293	8.1101	70	1.281	1.242	3.8142	80
Mucin-5AC	21.357	30.351	82.068	80	23.57	26.275	79.275	80
Neutrophil gelatinase-associated **lipocalin**	890.003	377.015	1209.287	60	987.514	382.932	1196.446	60
Haptoglobin	235.945	172.752	493.549	70	93.727	69.867	223.935	90

**Table 4:** Basic abundance variation of S100 calcium-dependent family members collected via Weck-Cel cervical sponge and cervicovaginal lavage as determined by mass spectrometry.

	Endocervical Sponge Protein Statistics				Cervicovaginal Lavage Protein Statistics			
Protein	Mean(x10^3^)	Std. Dev.(x10^3^)	Range(x10^3^)	% detected+/−1SD	Mean(x10^3^)	Std. Dev.(x10^3^)	Range(x10^3^)	% detected+/−1SD
Protein S100-A11	62.399	45.932	141.864	70	33.758	27.81	88.6379	70
Protein S100-A12	18.473	13.393	44.43	70	18.443	36.623	120.441	90
Protein S100-A2	12.852	20.843	69.979	90	11.896	28.493	92.337	90
Protein S100-A4	16.043	9.08	28.764	80	2.999	2.053	7.085	80
Protein S100-A6	52.863	35.967	110.056	60	11.011	26.025	84.142	90
Protein S100-A7	767.982	1484	4813.468	90	2119	5086	16503.87	90
Protein S100-A8	441.943	453.114	1377.098	80	1662	1661	4861.71	80
Protein S100-A9	403.404	396.168	1193.128	80	1436	1422	4097.986	80
Protein S100-P	42.886	32.266	90.548	60	15.24	9.907	27.261	60

**Table 5:** Basic abundance variation of serpin family members collected via Weck-Cel cervical sponge and cervicovaginal lavage as determined by mass spectrometry.

	Endocervical Sponge Protein Statistics				Cervicovaginal Lavage Protein Statistics			
Protein	Mean(x10^3^)	Std. Dev.(x10^3^)	Range(x10^3^)	% detected+/−1SD	Mean(x10^3^)	Std. Dev.(x10^3^)	Range(x10^3^)	% detected+/−1SD
Serpin A3	118.648	170.619	538.164	90	34.907	30.373	104.239	90
Serpin A1	155.812	95.116	258.713	50	90.249	43.377	142.226	70
Serpin B1	124.37	93.076	295.518	90	179.639	96.185	304.937	70
Serpin B10	0.3494	0.5662	1.675	70	0.8092	1.068	3.596	90
Serpin B13	18.211	14.431	49.624	80	52.059	38.776	105.991	80
Serpin B3	266.189	175.054	601.724	80	846.637	718.602	2130.868	80
Serpin B4	14.907	12.029	36.131	80	22.71	18.636	60.732	80

**Table 6:** Basic abundance variation of immunoglobulins (heavy chain variants) collected via Weck-Cel cervical sponge and cervicovaginal lavage as determined by mass spectrometry.

	Endocervical Sponge Protein Statistics				Cervicovaginal Lavage Protein Statistics			
Protein	Mean(x10^3^)	Std. Dev.(x10^3^)	Range(x10^3^)	% detected+/−1SD	Mean(x10^3^)	Std. Dev.(x10^3^)	Range(x10^3^)	% detected+/−1SD
Ig alpha-1 chain C region	454.726	209.718	731.341	80	375.066	175.716	458.073	40
Ig gamma-1 chain C region	2055	858.716	2829	90	1958	925.825	3408.74	80
Ig gamma-2 chain C region	391.944	237.228	767.211	80	339.37	224.441	670.559	70
Ig gamma-3 chain C region	250.382	155.805	521.606	80	240.785	157.15	466.322	70
Ig gamma-4 chain C region	77.837	168.929	549.721	90	47.038	94.003	0.047038	90
Ig mu chain C region	90.023	90.497	323.739	90	44.541	42.229	140.992	80

Of the 37 identified antimicrobial factors examined here, seven factors (Cathepsin G, Lysozyme C, A2ML1, S100-A8, S100-A9, Serpin B3 and Serpin B13) were recovered at statistically higher amounts in CVL, and another seven different factors (Cystatin B, Mucin-16, Haptoglobin, S100-A4, S100-A6, S100-P, and Serpin A3) were recovered at statistically higher amounts in CER samples. Furthermore, twelve previously described anti-HIV factors were identified in both collection methods: SLPI, Neutrophil defensin-1, Lysozyme C, Cystatin A, DMBT1, and Mucin 5B, Lactotransferrin, Histone H1.5, Histone H2B, Cystatin B, Serpin A1, and Serpin A3. Based on these results, it is clear that the anatomical site of collection and/or method of collection can have a drastic impact on the quantity and type of mucosal factors identified particularly inflammatory and innate immune factors.

## Discussion

Mucosal secretions are commonly used to evaluate the expression of innate immune factors and genital immune activation status. This form of evaluation is important for determining the safety and efficacy of HIV-1 prevention technologies such as microbicides and vaccines [22]–[24]. This study is the first to employ a systems biology approach to qualify the potential factors captured using the two most common sample collection methods in the FGT. Here we found that, each site sampled by its own collection method enriches for different inflammation pathways, and each method collects site-specific abundances of antimicrobial factors including anti-HIV-1 factors.

Immune activation has been identified as a major determinant of HIV-infection risk and the monitoring of these processes will be very important for therapeutic design. These results indicated that specific inflammation pathways were enriched in CER compared to CVL, which included the acute phase response and LXR/RXR activation pathways. These two pathways play significant roles in infection and/or inflammation events. The acute phase response is a major contributor to early-stage resistance against HIV-1 infection [35] and has been shown to be specifically up regulated in HIV-resistant individuals [11]. The LXR/RXR activation pathway causes alterations in lipoprotein metabolism to provide immediate protection to the host from potential damage that may occur during the acute stage of infection. Together, these pathways are meant to provide protection against microbes using non-specific defense mechanisms of the innate immune system. This is consistent with the fact that CER samples represent more of the endocervical secretions, and these soluble mediators may act as a barrier to prevent the invasion of pathogenic organisms into the relatively sterile environment of the uterus. In contrast, the proteins found to be significantly overabundant in CVL were involved in pathways related to the innate immune response. The top two enriched pathways were the role of IL-17A in psoriasis pathway and the complement system pathway. IL-17A plays an essential role in inducing an epithelially-derived antimicrobial response against extracellular pathogens [40]. It has been shown to increase the expression of skin derived antimicrobial peptides including β-defensins, Psoriasin (S100A7) and Calprotectin (S100A8/A9) in keratinocytes [40]. The complement system pathway, a well-known and understood component of the innate immune system, involves a cascade of enzyme activations that bridge the innate and acquired immune systems [41], [42]. This is consistent with the fact that CVL’s represent more proteins from the vaginal vault, which is in constant contact with commensal bacteria and requires these responses to maintain a healthy mucosal barrier. Clearly these collection methods impose a bias to enriched inflammation pathways as they sample specific compartments differentially. This adds yet another layer to the rationale that it is critical to sample both the upper and the lower compartments of the FGT to provide the most comprehensive representation of inflammation responses especially when evaluating mucosal therapeutics.

This proteomic study identified many factors with known HIV inhibitory properties in different mucosal secretions of the FGT, such factors include SLPI, Cystatin A & B, Lactotransferrin, Mucin 5B, Neutrophil defensin 1, Lysozyme C, DMBT1, Histones, Serpin A3 and A1, Calprotectin (S100 A8/A9), and mucosal IgA. Each of these anti-HIV factors were recovered in both CVL and CER; although abundances between compartments varied, with Neutrophil defensin-1, Lysozyme C, Calprotectin and Cystatin A found higher in CVL; Cystatin B and Serpin A3 found higher in CER; and Lactotransferrin, Histone H1.5, Histone H2B, Serpin A1, DMBT1, Mucin 5B and SLPI found in roughly equal amounts regardless of sampling site. The differences in abundances are likely demonstrating a compartmentalized difference in secreted immune function or sampling technique specificity. For example, CVL collects secretions primarily from the lower FGT which consists of the vaginal vault and ectocervix. This compartment is at the forefront of immunological defence, as it is met with the physical stress of sexual intercourse and the constant exposure to commensal microbiota and invasive pathogens [43]. Protection is conferred by a multilayered epithelium as well as numerous immune cells housed within the strata of these layers and submucosa. Conversely, CER samples are designed to collect secretions from the upper FGT, the endocervix and the endometrium. The upper FGT differs from the lower FGT, as its epithelium only consists of a single layer of columnar epithelial cells leaving it even more vulnerable to pathogens. Thus, the upper FGT has different immune mechanisms to compensate for its vulnerability [44]. These differences have been shown through studies examining immune factor expression with the ectocervix secreting the majority of proteins involved in innate immunity such as antiproteases, complement components and antimicrobial factors, while the endometrium secretes proteins mainly involved in tissue development [25]. Interestingly, of the three tissues examined, the endometrium produced many factors, which promote HIV infectivity. This compartmentalized difference is reinforced by the findings of our study which found an overlap in the immune factors collected by CVL and those that are known to be predominant immune factors expressed in the ectocervix [25]. However, Burgener *et al*.’s study also showed specific factors such as SLPI to be highest in abundance in endocervical tissue, whereas our study did not find a significant difference in CVL versus CER. Reasons for such results may be due to: sample cross contamination between CVL and CER collection, for instance cervical sponges may also collect secretions from the lower FGT upon removal or secretions from the upper FGT may be present in the lower FGT through natural process, or it may be due to certain factors being overexpressed in the tissue itself which is not reflected in the secretions examined. This provides evidence that both sampling techniques should ideally be used when measuring mucosal immune factors.

A previous study that specifically examined sampling techniques including CVL, vaginal swabs and endocervical swabs determined that secretions collected by CVL have higher anti-*Escherichia coli* activity over swabs, and both endocervical/vaginal swabs and CVL have comparable anti-HIV-1 activity [23]. This antimicrobial activity found in the secretions collected by endocervical/vaginal swabs and CVL is supported by the results of this study as there were a multitude of antimicrobial factors recovered and identified via both CER and CVL collection methods. The presence of high abundance antimicrobials such as S100 A8/A9, Lysozyme C, SLPI, and Defensins in CVL likely had contributing effects to its anti-*E. coli* activity, although antimicrobial and antiviral activity was not assessed in this study. Therefore, sampling techniques may have an impact on the measured antiviral response against HIV and other FGT infections; this may have important implications for studies and trials that monitor this activity.

It should be noted that there are other non-biological considerations for each collection method. For instance, CVL samples are more dilute than CER samples, which can make detection of low abundance proteins more challenging using mass spectrometry as the detection method. However, CVL provides a large sample volume for testing, typically recovers higher total protein content (assuming a 10 mL wash), and does not require an elution step as with sponges, which could result in protein loss. CER samples are more concentrated, sample more proteins from the upper FGT, but take longer to acquire and have smaller volumes with which to work. Another caveat of our study is the collection methods themselves, as noted above there are differences between swabs and lavages. A vaginal swab would have allowed for a more accurate comparison to an endocervical swab as it would have been a more accurate representation of the lower FGT than a lavage. Yet, the primary goal of our study was to examine the most commonly used sampling techniques of the female genital tract’s mucosa, particularly those that are used in clinical trials. Therefore, we chose to examine cervicovaginal lavage as it is the most highly used method to sample the mucosa of the female genital tract, followed by the endocervical sponge, therefore we felt it was more relevant to study those two collection methods.

There were some limitations to this study that warrant consideration. Although this proteomics technique was able to identify 385 unique proteins, other important immune factors such as cytokines and chemokines were not well represented in the analysis. This is likely due to their low abundance in mucosal secretions, which was below the detection threshold of the mass spectrometer. Therefore, we cannot comment on the impact of sample methodology on these factors. Further studies will also be needed to better dissociate between the type of collection method used and the site of collection to allow better localization of where specific innate factors are being secreted and from what cell types. Still, this study provides foundational information as it compares the most commonly used techniques and demonstrates that each collection method varies considerably in both types of mucosal factors collected as well as their abundances.

In summary, CER samples collected nearly twice as many unique proteins as CVL (111 vs. 61), yet each method still collected method/site-specific abundances of inflammatory pathways and antimicrobial factors. Therefore, neither the CER nor the CVL collection method is superior to the other, but together provide a more comprehensive scope of the immune mediators secreted by both the upper and lower compartments of the FGT. As it is still unclear which factors are important in immune activation of the FGT and/or HIV susceptibility, future research should be prudent to utilize both sampling techniques to obtain the greatest recovery and most accurate measurement of the biomarkers naturally produced in the FGT. This information should be taken into consideration for studies evaluating mucosal immune responses in the development of mucosal therapeutics such as microbicides and vaccines or other aspects of reproductive immunology.

## Supporting Information

Table S1 **All proteins identified and quantified via Weck-Cel cervical sponge and cervicovaginal lavage collection methods.**
(XLSX)

Table S2 **Basic abundance variation of all proteins collected via Weck-Cel cervical sponge and cervicovaginal lavage as determined by mass spectrometry.**
(XLSX)
